# Prevalence of Severe Hypercholesterolemia and Familial Hypercholesterolemia Phenotype in Patients with Acute Coronary Syndrome

**DOI:** 10.3390/medicina61040681

**Published:** 2025-04-07

**Authors:** Urtė Aliošaitienė, Aleksandras Laucevičius, Urtė Smailytė, Egidija Rinkūnienė, Roma Puronaitė, Jūratė Barysienė, Žaneta Petrulionienė

**Affiliations:** 1 Faculty of Medicine, Vilnius University, 01513 Vilnius, Lithuania; urtesmailyte@gmail.com (U.S.); egidija.rinkuniene@santa.lt (E.R.); jurate.barysiene@santa.lt (J.B.); zaneta.petrulioniene@santa.lt (Ž.P.); 2 Clinic of Cardiac and Vascular Diseases, Vilnius University Hospital Santaros Klinikos, 01513 Vilnius, Lithuania; 3 State Research Institute Centre for Innovative Medicine, 01513 Vilnius, Lithuania; aleksandras.laucevicius@santa.lt; 4 Center of Informatics and Development, Vilnius University Hospital Santaros Klinikos, 01513 Vilnius, Lithuania

**Keywords:** severe hypercholesterolemia, familial hypercholesterolemia, atherosclerotic cardiovascular disease, acute coronary syndrome

## Abstract

*Background and Objectives*: Atherosclerotic cardiovascular disease is one of the most common causes of death and disability around the world. Hypercholesterolemia is an established and widely prevalent risk factor; however, the prevalence of severe hypercholesterolemia (which is characteristic for familial hypercholesterolemia) has been studied far less. The aim of this study was to determine the prevalence of severe hypercholesterolemia among patients with acute coronary syndrome. *Materials and Methods*: A retrospective study of patients hospitalised at Vilnius University Hospital Santaros Klinikos due to acute coronary syndrome was performed. Data were attained from an electronic medical history database. Data such as sex, age, cardiovascular risk factors (hypertension, diabetes) and low-density cholesterol results were collected. Severe hypercholesterolemia was defined as low-density lipoprotein cholesterol levels ≥ 4.9 mmol/L. *Results*: A total of 34,669 patients were included in this study (12,115 females (34.9%) and 22554 (65.1%) males, *p* < 0.001). The median age of the entire study population was 67 years. A total of 3434 patients (9.9%) had severe hypercholesterolemia, 371 (1.1%) patients met the criteria for phenotypically probable familial hypercholesterolemia, and 36 (0.1%) patients presented with phenotypically definite familial hypercholesterolemia. The most common concomitant risk factor in this study was arterial hypertension, which was found in 48% of patients. *Conclusions*: Based on the results of this study, severe hypercholesterolemia is prevalent among patients with acute coronary syndrome, with as many as 9.9% of patients presenting with severe hypercholesterolemia at the time of hospitalisation. The definite familial hypercholesterolemia phenotype is scarcer, with prevalence reaching 0.1% of patients with acute coronary syndrome.

## 1. Introduction

Cardiovascular diseases remain the most common cause of death worldwide [1]. Despite the progress of cardiovascular medicine, which led to the declining mortality rate, due to the growing population, the aging of society, and the consequent increase in the number of polymorbid patients, the number of patients with cardiovascular diseases is still increasing. One of the most common types of cardiovascular diseases is atherosclerotic cardiovascular disease (ASCVD), which can manifest as acute coronary syndrome (ACS). ASCVD causes not only cause a significant loss of DALYs (Disability-Adjusted Life Years), but also, oftentimes, remains deadly, as patients who suffer a non-fatal cardiovascular event are at high risk of a repeated event in the first year (and especially in the first month) after the ACS [2,3]. Patients with ASCVD often are polymorbid and/or have worse quality of life. Having ASCVD is even associated with a higher risk of depression [4]. Many risk factors for atherosclerosis (and by extent for ASCVD) have been identified, from modifiable (lifestyle) risk factors, such as smoking, arterial hypertension (AH), diabetes mellitus (DM) and low physical activity levels, to non-modifiable factors, such as genetic predisposition, age and sex; in recent years, even novel risk factors, such as usage of alcohol, high lipoprotein a (Lp(a)) concentrations, sleep disorders and air pollution [5,6]. Yet, dyslipidaemia, which plays perhaps the most important role in the progression of atherosclerosis, remains one of the most common risk factors, although its prevalence varies depending on the region [7]. Dyslipidaemia does not usually cause any symptoms and, therefore, remains an underestimated and undertreated condition. Furthermore, there are several types of dyslipidaemias; however, one of the most common forms is hypercholesterolemia, usually defined as either increase of total cholesterol (TC) or low-density lipoprotein cholesterol (LDL-C). Severe hypercholesterolemia (SH) is defined as an increase of LDL-C ≥ 4.9 mmol/L [8]. SH is an important cardiovascular risk factor, as it is associated with higher cardiovascular risk; not to mention it, requires more aggressive lipid-lowering treatment [9]. In addition, recent studies suggest that LDL-C plays a role in development of not only atherosclerosis, but also other diseases, for instance, hepatosteatosis, osteoporosis and others [10]. As SH has only started gaining attention, studies on its prevalence in many regions are lacking and the Baltics are no exception. Moreover, despite associations of SH and atherosclerosis, data on its prevalence among patients with cardiovascular diseases are also limited. SH, like other forms of dyslipidaemias, can be classified as primary or secondary (caused by such conditions as nephrotic syndrome, cholestasis and hypothyroidism) [11]. On the contrary, primary causes of SH are genetic—the most common among them being familial hypercholesterolemia (FH) [12]. The assumed prevalence of FH is, on average, 1:200–1:250; however, it remains unknown in the vast majority of the world, including Lithuania, as it is frequently underdiagnosed [13]. Patients with FH are at higher risk of not only increased LDL-C, but also of experiencing a cardiovascular event, which, in more severe cases, can manifest prematurely (at a younger age than in the general population) [14]. Therefore, increasing the scope of FH diagnostics is vital. Unfortunately, there still is no golden standard for the screening for FH. Several countries have implanted universal screening programmes for FH, and although more cases tend to be found in these countries, the cost–benefit ratio remains to be controversial. Furthermore, studies show that there is a lack of knowledge about FH not only among patients, but also in the medical community [15]. In light of the gaps in knowledge on both the prevalence of severe hypercholesterolemia, especially among patients with acute coronary syndrome, and familial hypercholesterolemia, more research is urgently needed. Therefore, the aim of this study was to determine the prevalence of SH in patients treated for ACS in Vilnius university hospital Santaros clinics (VUHSC).

## 2. Materials and Methods

The study was approved by the Vilnius (Lithuania) regional bioethics committee (permit number 158200-18/5-1010-538, issued 18 May 2018). A retrospective search of VUHSC electronic patient database was performed. All patients hospitalised due to ACS between 2005 and 2023 were selected for the study. Demographic data, such as age (in years) at the time of the hospitalisation and sex (males and females), as well as clinical data, including the results of lipid panels and documented risk factors (i.e., hypertension, diabetes mellitus), were collected. For the analysis, patients were pooled into groups based on their phenotype: The SH group included all patients with LDL-C ≥ 4.9 mmol/L, the phenotypically probable FH group included all patients with LDL-C ≥ 6.5 mmol/L and the phenotypically definite FH group included all patients with LDL-C ≥ 8.5 mmol/L. LDL-C cut-off values, used for assessment of FH probability, were chosen based on Dutch Lipid Clinical Network (DLCN) criteria. Statistical comparisons were made between the selected group and all the remaining patients who were not included in the analysed group.

### Statistical Analysis

All statistical analysis was performed using the R (v. 4.0.4) program package. The mean, standard deviation (SD), quartiles (Q1 and Q3), median and available number of observations of the quantitative variables are presented. Categorical variables are presented as the absolute amount and the percentage. In order to test hypotheses for between two groups comparison of the quantitative variables, Student’s *t*-test or nonparametric Mann–Whitney U test was used as appropriate. Normality was tested using the Shapiro–Wilks test. In order to test hypotheses for between-group comparison of the categorical variables, Pearson’s Chi-Square or Fisher ‘s exact tests were used as appropriate. A *p*-value less than 0.05 was considered significant.

## 3. Results

A total of 34,669 patients were enrolled in this study; among them, 12,115 (34.9%) were females and 22,554 (65.1%) were males, *p* < 0.001. The median age of the entire study population was 67 years (Q1 = 58, Q3 = 76, IQR = 18). The median age was significantly higher among females (73 years (Q1 = 65, Q3 = 79, IQR = 14)) compared to males (64 years (Q1 = 56, Q3 = 73, IQR = 17)); *p* < 0.001. The general characteristics of the study population are presented in Table 1. In all categories, the differences among sexes were statistically significant.

**Figure 1:**
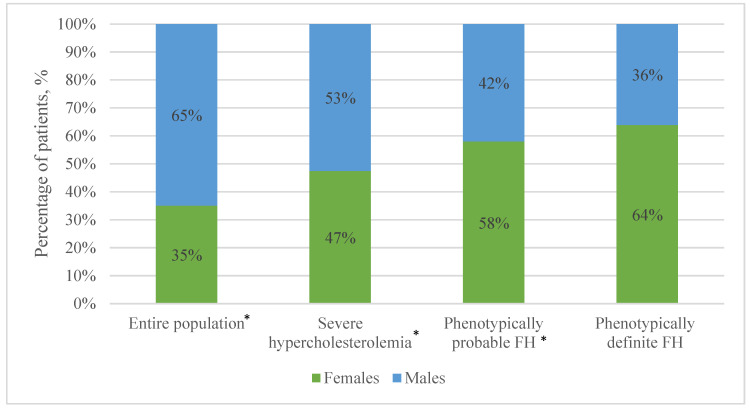
Distribution of sexes in patient groups. FH—familial hypercholesterolemia. * *p* < 0.05; statistically significant difference in proportion of sexes in a patient group.

A total of 3434 patients (9.9%) were diagnosed with SH. Among these patients, 1805 (8.0%) were males and 1629 (13.4%) were females. Females comprised 47.4% of the group and males comprised 52.6%. The median age of this subgroup was 66 years (Q1 = 57, Q3 = 74, IQR = 17) and was statistically significantly different compared to patients who did not have SH (median age 68 years (Q1 = 59, Q3 = 76, IQR = 1)). The prevalence of hypertension in this sub-group was 46.5%, while in patients without SH, it was 48.1%, *p* = 0.072. However, the difference in the prevalence of diabetes and the combination of diabetes with hypertension between the SH group and patients without SH was statistically significant, (9% vs. 12% and 7% vs. 10%, respectively). Distribution of sexes in different patient groups is presented in Figure 1. 

**Figure 2:**
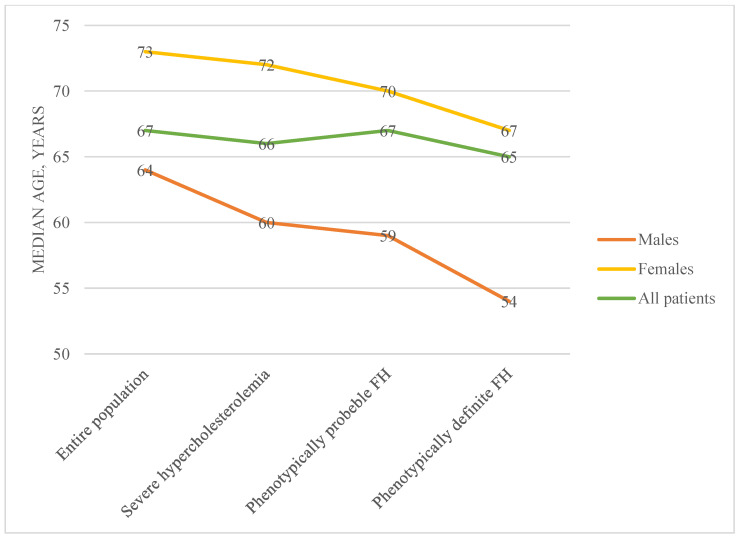
Median age in different patient groups. FH—familial hypercholesterolemia.

A total of 371 (1.1%) patients met the criteria for phenotypically probable FH. The median age of this subgroup was 67 years (Q1 = 58, Q3 = 75, IQR = 17); no statistical significance was found in comparison to patients who did not meet the criteria for phenotypically probable FH. Of this patient group, 57.7% was female. No statistically significant differences in the prevalence of cardiovascular risk factors were found when females of this sub-group were compared to females who were not included in this group. However, only 5.1% of males with phenotypically probable FH had diabetes, in contrast to males who did not have phenotypically probable FH (10%), *p* < 0.005. Changes in median age in different patient groups is presented in Figure 2.

**Figure 3:**
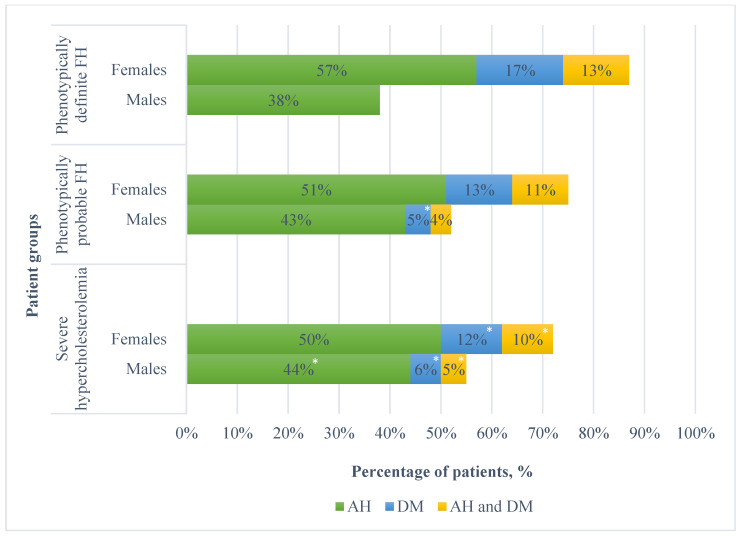
Prevalence of cardiovascular risk factors by sex in different patient groups. FH—familial hypercholesterolemia; AH—arterial hypertension; DM—diabetes mellitus. * *p* < 0.05; statistically significant difference in prevalence of cardiovascular risk factor between patients in an analysed group and remaining study subjects.

A total of 36 patients presented with phenotypically definite FH. The mean age in this group was 64 (±12.3); however, there was no statistically significant difference when compared to patients who did not have phenotypically definite FH. Of this sub-group, 63.9% was female. There were no statistically significant differences in the prevalence of cardiovascular risk factors between this group (for both sexes) and patients without phenotypically definite FH. Prevalence of various cardiovascular risk factors in different patient groups is presented in Figure 3. 

## 4. Discussion

Despite the advances in cardiology and increasing availability of medical services, ACS remains a frequent cause of death worldwide. Therefore, cardiovascular risk management is an important part of lowering ASCVD-related mortality. Patients that have already been diagnosed with ACS are of special importance, as far as cardiovascular risk factors management is concerned, since they are in the very high-risk group. Dyslipidaemia is still one of the most common cardiovascular risk factors, and hypercholesterolemia remains of the most common types of dyslipidaemia [7]. Although the prevalence of dyslipidaemia varies depending on the region, it tends to be higher in urbanised areas and where the western diet is of increasing popularity [7]. Furthermore, the prevalence increases with age; therefore, it is higher in ageing populations. In Lithuania, the prevalence of dyslipidaemia is very high—according to previous research, the prevalence is as high as 90% in the middle-aged population [16]. In this study, the median LDL-C was 3.4 mmol/L. However, the lipid panels in this study were performed for patients in the acute phase of the ACS, which is known to cause lowering of LDL-c levels by as much as 1.7–39% [17,18]. This phenomenon could also cause a lower LDL-c median of the patients in this study than could be anticipated. Nonetheless, as the median is higher than the target values recommended by European Society of Cardiology guidelines, it highlights the suboptimal reach of cardiovascular prevention.

In terms of cardiovascular risk management, it is also important to control LDL-C levels, especially among SH patients. Several medication options are currently available for the management of hypercholesterolemia—the first line of treatment is statins (hydroxy-methyl-glutaryl-coenzyme A (HMG-CoA) reductase inhibitors), while adding ezetimibe and Proprotein convertase subtilisin/kexin type 9 (PCSK9) inhibitors are recommended when target LDL-c levels cannot be reached with statins alone [19]. It is important to not only initiate lipid-lowering therapy for patients with hypercholesterolemia, but also to ensure patient’s cooperation and adherence to therapy. It is known that intensified patient care can improve the patient’s adherence to therapy and, therefore, the patient’s lipid profile [20]. However, research is needed to better understand the impact of psychological determinants and their effect on adherence to therapy, especially in patients with FH. Lifestyle changes should also be considered. However, while exercise has numerous positive effects on general health of the patient, the effect it has on LDL-c levels is inconsistent and findings from studies on the topic are varying [21]. However, regular physical activity could be beneficial in reducing cardiovascular risk through other pathways than dyslipidaemia and, therefore, should not be overlooked.

SH causes an increase in cardiovascular risk even in the absence of other risk factors and is, therefore, a condition that should not be underestimated [22]. However, studies on prevalence of SH are still sparse, particularly in the Baltic region. There are also, to the best of our knowledge, no studies defining the prevalence of SH among patients with ASCVD. According to existing data, the prevalence of SH varies between 4 and 13% depending on the region, population age and other population-defining characters [23,24,25,26].

In this study, the prevalence of SH was determined to be 9.9% among patients with ACS—such a prevalence is in accordance with other studies; however, it is lower than to be expected, as all patients in this study have been diagnosed with ASCVD and the vast majority are of an older age and from a very high cardiovascular risk region, with a high prevalence of dyslipidaemias.

SH is a symptom and not a disease. There are multiple causes of secondary SH—such as cholestasis, nephrotic syndrome or the usage of certain medications. Therefore, careful assessment of patient’s history, a thorough examination and appropriate diagnostic tests are of great importance. On the other hand, the most common primary cause of SH is FH. However, despite the recent increase in data on FH, the disease remains underdiagnosed [13]. Only a few countries have high detection rates, and it is most likely related to the lack of universal screening programmes. However, as universal screening programmes are difficult to implement, other detection tools are important. Namely, as patients with FH may present with SH, it is fitting to search for FH in SH populations. Furthermore, FH is more prevalent among patients with ASCVD. However, in this study, phenotypically definite FH was found in only 36 patients, or 0.1% of the study population. It should be considered that, due to the design of the study, not all criteria used for diagnosing FH (for instance, accurate personal and familial anamnesis of ASCVD as well as hypercholesterolemia, history of xanthomas, lipoid arcus, etc.) could be taken into account and, therefore, it is very likely that the real prevalence of FH in this study could be higher. In comparison, the prevalence of phenotypically probable FH, defined by LDL-C ≥ 6.5 mmol/L, was 1.1%. A similar study in Poland found the prevalence of such LDL-C increase to be 4.34% [27]. Therefore, the prevalence of FH phenotypes in this study is lower than could be anticipated.

Apart from hypercholesterolemia, this study also evaluated the prevalence of other risk factors, namely, hypertension and diabetes mellitus, in patients with ACS. The prevalence of hypertension in this study was high, with 48% of included patients having it at the time of hospitalisation. However, this is not unexpected, as other studies have found similar results—for example, in the National Registry of Myocardial Infarction (NRMI) study, the prevalence of hypertension was 52% [28]. This shows that, despite improving prevention programmes and interventions, hypertension remains a widely prevalent cardiovascular risk factor, especially among patients with ASCVD. Furthermore, the prevalence of diabetes in this study was 12%, which is also in accordance with the prevalence in the general population [29]. It should be noted that recent studies show that the combination of diabetes and SH is more dangerous than SH alone, as patients with both of these factors are at a higher risk of a cardiovascular event [30]. Therefore, these patients should be considered with special carefulness.

Interestingly, in this study, females were the majority in the phenotypically probable and phenotypically definite FH groups, as well as making up 47% of the SH group, despite being the minority in the entire study population (comprising only 34.9%). This could be partially explained by the older median age of females in this study compared to males, as a higher prevalence of dyslipidaemias is observed in older populations. Furthermore, older females are more likely to have hypercholesterolemia due to hormonal changes caused by menopause [31]. However, recent studies suggest that women are possibly more affected by dyslipidaemias and, perhaps, FH is more prevalent among females [32]. A study in Poland also found a similar trend in FH prevalence among sexes, but since studies on this topic are still scarce, further research is required [27].

### Limitations of the Study

Despite the novelty of the results from this study, it has several limitations. First of all, due to the retrospective/cross-sectional design of this study, only the correlations (and not reasoning) of the findings can be evaluated. Additionally, due to data availability limitations, some important factors (for example, smoking, obesity, participation in exercise, familial history of cardiovascular events, personal medical history, lipid-lowering therapy and influence of other medications, as well as adherence to therapy) could not be evaluated. Also, the research is performed from an electronic database, which may lead to biases or potential loss/misclassification of data, however, due to large data sample it is unlikely to affect the overall results. Lastly, this research was performed in only one tertiary hospital and therefore the generalization of these results for other population should be used with consideration.

## 5. Conclusions

The prevalence of severe hypercholesterolemia in patients with acute coronary syndrome is 9.9%. The prevalence is higher among females (13.4%, N = 1629) than in males (8.0%, N = 1805)

The prevalence of phenotypically probable FH was 1.1%, whereas phenotypically definite FH was found in 0.1% of patients with acute coronary syndrome.

The prevalence of arterial hypertension among patients with acute coronary syndrome was 48% and the prevalence of diabetes mellitus was 11.8%, whereas the combination of both cardiovascular risk factors was prevalent in 9.3% of patients with acute coronary syndrome. In this study, females were found to have a higher prevalence of cardiovascular risk factors.

Women with phenotypically definite FH were diagnosed with ACS 6 years earlier, and men 10 years earlier, than the overall study population (respectively, women and men).

## Figures and Tables

**Table 1:** General characteristics of the study population.

	Study Population	Males	Females	*p* Value
**Total (N)**	34,669	22,554 (65.1%)	12,115 (34.9%)	**<0.001 ^†^**
LDL-C ≥ 4.9 mmol/L (N, %)	3434 (9.9%)	1805 (8.0%)	1629 (13.4%)	<0.001 *
LDL-C ≥ 6.5 mmol/L (N, %)	371 (1.1%)	157 (0.7%)	214 (1.8%)	<0.001 *
LDL-C ≥ 8.5 mmol/L (N, %)	36 (0.1%)	13 (0.1%)	23 (0.2%)	<0.001 *
DM (N, %)	4081 (11.8%)	2244 (9.9%)	1837 (15.2%)	<0.001 *
AH (N, %)	16,627 (48.0%)	10,458 (46.4%)	6169 (50.9%)	<0.001 *
DM and AH (N, %)	3229 (9.3%)	1743 (7.7%)	1486 (12.3%)	<0.001 *
Median age (years)	67	64	73	<0.001 *
Median LDL-C (mmol/L/)	3.4	3.3	3.5	<0.001 *

LDL—low density lipoprotein cholesterol; DM—diabetes mellitus; AH—arterial hypertension. ^†^ Statistically significant difference in proportion between sexes in a patient group. * Statistically significant difference between sexes.

## Data Availability

The raw data supporting the conclusions of this article will be made available by the authors on request.
